# Safety and efficacy evaluation of halicin as an effective drug for inhibiting intestinal infections

**DOI:** 10.3389/fphar.2024.1389293

**Published:** 2024-05-09

**Authors:** Maolu Zhang, Shuqian Lin, Lianquan Han, Jiaming Zhang, Shaoning Liu, Xiuzhen Yang, Ruiming Wang, Xiaohui Yang, Yunpeng Yi

**Affiliations:** ^1^ State Key Laboratory of Biobased Material and Green Papermaking (LBMP), Qilu University of Technology (Shandong Academy of Sciences), Jinan, Shandong, China; ^2^ Shandong Provincial Animal and Poultry Green Health Products Creation Engineering Laboratory, Institute of Poultry Science, Shandong Academy of Agricultural Science, Jinan, Shandong, China; ^3^ Animal Products Quality and Safety Center of Shandong Province, Jinan, Shandong, China

**Keywords:** halicin, antimicrobial activities, ames test, safety evaluation, *Clostridium perfringens*

## Abstract

Halicin, the first antibacterial agent discovered by artificial intelligence, exerts broad-spectrum antibacterial effects and has a unique structure. Our study found that halicin had a good inhibitory effect on clinical isolates of drug-resistant strains and *Clostridium perfringens* (*C. perfringens*). The safety of halicin was evaluated by acute oral toxicity, genotoxicity and subchronic toxicity studies. The results of acute toxicity test indicated that halicin, as a low-toxicity compound, had an LD_50_ of 2018.3 mg/kg. The results of sperm malformation, bone marrow chromosome aberration and cell micronucleus tests showed that halicin had no obvious genotoxicity. However, the results of the 90-day subchronic toxicity test indicated that the test rats exhibited weight loss and slight renal inflammation at a high dose of 201.8 mg/kg. Teratogenicity of zebrafish embryos showed that halicin had no significant teratogenicity. Analysis of intestinal microbiota showed that halicin had a significant effect on the intestinal microbial composition, but caused a faster recovery. Furthermore, drug metabolism experiments showed that halicin was poorly absorbed and quickly eliminated *in vivo*. Our study found that halicin had a good therapeutic effect on intestinal infection model of *C*. *perfringens*. These results show the feasibility of developing oral halicin as a clinical candidate drug for treating intestinal infections.

## 1 Introduction

Since their discovery, antibiotics, have played a crucial role in saving countless lives, and they have become the most effective tools for treating bacterial infections. However, the widespread use of antibiotics has led to an alarming increase in antibiotic-resistant bacteria, with the rapid spread and accumulation of resistance genes among bacterial populations (Crits-Christoph et al., 2022). Unfortunately, the development speed of new antibiotics is significantly lower than the emergence rate of antibiotic-resistant strains. Even antibiotics once hailed as the ‘last line of defense’ such as vancomycin and colistin, have now been found to have strains resistant to them (Gao et al., 2016). This poses significant challenges in the clinical treatment of bacterial infections. Currently, effective approaches for treating infections caused by antibiotic-resistant bacteria include the development of new antibiotics with diverse mechanisms of action, combination therapies, and antibiotic rotations (Mitcheltree et al., 2021; Selvarajan et al., 2022; Shukla et al., 2023).

In the era of rapid development of science and technology, artificial intelligence (AI) is significantly impacting various fields, especially new drug development and screening (Gupta et al., 2021). AI excels in processing and analyzing complex data, streamlining the drug development process by shortening research times, cutting costs, and enhancing success rates. During drug screening, AI algorithms efficiently search through extensive compound libraries to identify active candidates, improving both the speed and precision of this phase (Deng et al., 2021). To address the problem of drug resistance, AI offers new ways for finding antibiotics with novel action mechanisms. One example is the discovery of halicin, a unique broad-spectrum antibiotic, which was identified using AI from over 6,000 compounds in the Drug Repurposing Hub (Stokes et al., 2020).

Halicin has garnered substantial attention in recent years because of its potential therapeutic applications. As a potential broad-spectrum antibacterial agent, halicin showed remarkable efficacy against both Gram-positive and Gram-negative bacteria (Stokes et al., 2020; Booq et al., 2021; Higashihira et al., 2022). Halicin has a lower likelihood of inducing bacterial resistance than traditional antibiotics. This may be due to its unique mode of action or other factors, making it a promising candidate for further study. It is particularly noted for its effectiveness against *Clostridioides difficile* (*C. difficile*) and *A. baumannii* (*Acinetobacter baumannii*). *In vitro* studies showed that halicin could effectively inhibit the growth of drug-resistant strains. Meanwhile, halicin had antibacterial activity against *C. difficile in vivo* (Stokes et al., 2020).

Recently, it has been reported that halicin has exceptional membrane-disrupting effects on *Staphylococcus aureus* (*S. aureus*) (Higashihira et al., 2022). Furthermore, when combined with vancomycin, it exhibited synergistic effects and effectively inhibited the growth of *Enterococcus faecium* (Hussain et al., 2022). These findings highlight the significant research value of halicin in treating infections caused by antibiotic-resistant bacteria. However, current research on halicin mainly focuses on its remarkable antibacterial effect, and there is a lack of systematic evaluation of its drug properties. Additionally, studies on the toxicity of halicin and its potential adverse effects on humans are lacking.

Therefore, we conducted a preliminary pharmacodynamic and toxicological evaluation of halicin. We assessed its antibacterial activity against both common bacteria and clinically isolated drug-resistant strains, and conducted initial safety and efficacy assessments. To further investigate the effect of halicin on the gut microbiota, we collected and analyzed fecal samples from mice treated with halicin, and performed gut microbiota analysis. In addition, a model of intestinal infection with *C. perfringens* was used to evaluate the pharmacodynamics of halicin *in vivo*.

## 2 Materials and methods

### 2.1 Strains and culture conditions

All bacterial strains used in this experiment were stored at −80°C in culture media containing 25% glycerol. The bacteria were revived in LB agar medium and prepared for experiments. The clinical isolates used in this study were obtained from the Chinese Academy of Agricultural Sciences. Detailed descriptions of these bacteria can be found in Supplementary Table S1.

### 2.2 Reagents and materials

Halicin was synthesized and identified using NMR in our laboratory (Purity, 99.5%). Carboxymethyl cellulose sodium (CMC-Na) was purchased from Shanghai Chemical Reagent Co., Ltd. Cyclophosphamide was purchased from Beyotime Biotechnology Co., Ltd. Ciprofloxacin was purchased from Energy Chemical Shanghai Co., Ltd.

### 2.3 Minimum inhibitory concentration (MIC)

The test strains were inoculated on the corresponding solid medium and incubated overnight at 37°C. A single colony was picked from the medium and inoculated into Luria Broth (LB), and then incubated overnight at 37°C with shaking at 220 rpm. The bacterial suspension was diluted to 0.5 McFarland turbidity with Mueller Hinton broth (MHB), and then dilute 100 times for later use. Halicin was added to the 96-well plate, and diluted it to concentrations of 128, 64, 32, 16, 8, 4, 2, 1, 0.5, 0.25, 0.125, and 0 μg/mL by gradient. The 96-well plate was incubated at 37°C for 16 h, followed by observing and recording the results. The MIC was determined as the concentration at which there was no visible colony growth was observed.

### 2.4 Continuous induction of antimicrobial resistance in bacteria

According to the determination method of MIC, the bacterial cells were diluted to different concentrations and mixed with halicin, then incubated overnight. Next day, the sample close to the MIC were selected for re-culture, the concentration was adjusted to 1 × 10^6^ CFU/mL, and the above steps were repeated. Meanwhile, the strains were preserved daily, and the changes of MIC were recorded.

### 2.5 Growth kinetics curve

Briefly, *Escherichia coli* (ATCC 25922), *Salmonella* (CVCC 3377), *S. aureus* (ATCC 29213), *Klebsiella pneumoniae* (ATCC 4352) and *Proteus* (CMCC (B) 49027) (1 × 10^6^ CFU/mL) were transferred into MHB. Different concentrations of halicin were added to achieve final concentrations of 4 ×, 2 ×, 1 ×, 1/2 ×, and 0 ×MIC. The optical density at 600 nm was determined.

### 2.6 Ames assay

After dissolving 2 mg of halicin in 1 mL DMSO, the solution was serially diluted in distilled water. The resulting dilutions were mixed with melted top agar to obtain final halicin concentrations of 45, 22.5, 11.2, and 5.6 μg/plate. Positive controls were used with and without the mammalian microsomal enzyme test (S9). The Ames test was conducted as previously described with modifications (Mello Silva Oliveira et al., 2016; Wahab et al., 2018; Fan et al., 2022). The experiments were performed in triplicate.

### 2.7 Animal source and housing conditions

Both female and male Sprague Dawley (SD) rats and ICR mice were provided by the Shandong Pengyue Experimental Animal Breeding Center. All experimental animals were housed in standard rodent housing conditions, with a temperature of 20°C–24°C, a relative humidity of 55% ± 10%, and a 12/12-h light/dark photoperiod. They had free access to water and food. All animals underwent a 2-week quarantine period and a physical examination before being provided. Prior to the experiment, animals were allowed a 3-day adaptation period. All animals were fasted overnight before the administration of treatment but had free access to water.

### 2.8 Acute oral toxicity test in ICR mice

Fifty male and female ICR mice weighing 18–22 g were randomly divided into five groups to conduct an acute oral toxicity test and determine the LD_50_. The mice were given 4,000, 2000, 1,000, 500, and 0 mg/kg (control group) halicin in 0.1 mL per 10 g of body weight using the gavage method. Following administration, their health status was monitored for a period of 7 days. Survival rates and signs of toxicity were recorded. Furthermore, a post-mortem examination was conducted to observe any pathological changes in the organs of deceased animals (Wang et al., 2017).

### 2.9 Sperm abnormality test in mice

Fifty male ICR mice, aged 6–8 weeks and weighing 25 ± 2 g, were randomly divided into the following five groups (*n* = 10 mice per group): the high-dose group (1009.2 mg/kg), the medium-dose group (504.6 mg/kg), the low-dose group (252.3 mg/kg), the negative control group (0 mg/kg), and the positive control group (40 mg/kg cyclophosphamide). The mice in the experimental groups were orally administered halicin via gavage. The treatment was conducted every 24 h for five consecutive days. After 35 days from the initial administration, the mice were euthanized by cervical dislocation. Mouse spermatozoa were smeared and then stained using eosin staining solution (Beyotime, China). Under low magnification, non-overlapping areas with a uniform distribution were selected. High magnification was then used to observe and record the percentage of sperm abnormalities and the number of different types of abnormalities present (OECD, 2018; Dong et al., 2022).

### 2.10 Chromosomal aberration test in mice

Forty ICR mice, with a 1:1 male: female ratio weighing 18–22 g were selected. The dose group settings were the same as 2.9. The mice received three consecutive administrations, spaced 24 h apart. Two to 4 hours before euthanasia, the mice were treated with 2 mM colchicine through intraperitoneal injection. After euthanasia, the bilateral femurs were promptly extracted and cleansed of any blood. Following the guidelines for veterinary drug experiments, bone marrow cells were collected for slide preparation. Samples were stained using Giemsa staining solution (Beyotime, China). A total of 100 cells with well-separated chromosomes in the metaphase stage were randomly selected from each sample for observation. Chromosomal aberrations, such as chromosome or chromatid breaks and chromosome deletions, were documented (OECD, 2016; Akagi et al., 2023).

### 2.11 Micronucleus experiment of mammalian bone marrow cells

Fifty ICR mice (weight 18–22 g), were divided into the following five groups (*n* = 10 mice per group). The dose group settings were the same as 2.9. The mice received two consecutive doses, separated by 24 h. All medications were given through tube feeding with 0.5% CMC-Na as the solvent. Six hours after the second administration, the mice were sacrificed. Both femurs were removed, the bone marrow cells were washed with 0.2 mL of calf serum, and the tablets were smeared. Slides were smeared and then stained using eosin staining solution (Beyotime, China). Using a double-blind procedure, 1,000 polychromatic erythrocytes cells were observed from each mouse (OECD, 2016; Guo et al., 2021).

### 2.12 90-Day subchronic toxicity assay

Forty rats (weight 100 ± 10 g) were randomly divided into four groups (*n* = 10 rats per group). Each rat was housed in a separate cage. The experiment comprised three treatment groups and one negative control group (0.5% CMC-Na). The dosage concentrations for the treatment groups were determined based on the LD_50_ results obtained from the acute oral toxicity test. The high-dose group received a concentration of 201.8 mg/kg, the medium-dose group received 100.9 mg/kg, and the low-dose group received 50.5 mg/kg. Administration was conducted via oral gavage, with a dosage of 1 mL per 100 g of body weight, for a continuous period of 13 weeks. Throughout the study period, body weight, food intake, and water consumption were recorded daily. Additionally, clinical observations and behavioral assessments were conducted. On day 45, which marked the mid-term of the study, blood samples were collected from the rats through the jugular vein. To minimize external interference with the experimental results, the rats were subjected to a 16-h fasting period before blood collection. Whole blood samples were stored in EDTA anticoagulant tubes, and routine hematological parameters were analyzed using a Mindray hematology analyzer.

Clinical chemistry parameters were measured using the ELLIPSE (YSBAERT) clinical chemistry analyzer. At both the mid-term (Day 45) and the end of the experiment (Day 90), five rats from each group were euthanized by cervical dislocation for necropsy. The hearts, livers, spleens, lungs, and kidneys of the rats were embedded in paraffin, stained with eosin and hematoxylin, and observed under a microscope to assess the impact of the treatment on their organs (OECD, 2018; Guefack et al., 2022).

### 2.13 Zebrafish embryotoxicity test

Under controlled laboratory settings, wild-type zebrafish of strain AB were maintained under a 14/10-h light/dark photoperiod at a constant temperature of 27°C. Approximately 4–5 h prior to the embryotoxicity assay, zebrafish ova were harvested. We primarily utilized ova that had progressed to the blastula stage of embryonic development (OECD, 2013; Adam et al., 2021). Approximately 4–5 h after the fertilization of zebrafish oocytes, halicin was introduced into the culture medium. We employed a 32-well plate format, allocating 20 oocytes to each experimental group. Concentrations of halicin in the culture medium were 128, 64, 32, 16, and 8 μM. As a control, 0.2% DMSO was used. The cultures were maintained at a constant ambient temperature of 27°C. After drug administration, survival of the zebrafish embryos was recorded for 72 h. The LC_50_ of halicin on zebrafish was obtained by this method.

For embryonic malformation studies, groups of 20 zebrafish embryos each were treated with halicin. The concentrations of the drug were set at 4, 2, 1, and 0.5 μM, in accordance with established protocols for zebrafish embryo acute toxicity test. Observations were conducted at 24, 48, and 72 h after fertilization using a microscope. The embryonic assessment focused on six specific teratogenic endpoints: pericardial edema, yolk sac edema, cardiac malformations, morphological body shape deformities, tail deformities, and craniofacial anomalies, as well as otolith abnormalities. To minimize the impact of random variability, these experiments were performed in triplicate (OECD, 1998).

### 2.14 Microbiological analysis

Six-week-old BALB/c mice, with an equal distribution of males and females (*n* = 10 per group), were selected for the study. The mice were divided into three groups: control group, halicin group, and ciprofloxacin (CIP) group. Administration was conducted via oral gavage at a dose of 10 mg/kg. The treatment was given once daily, with a 24-h interval, for a continuous period of 5 days. Fecal samples were collected from the mice every day to analyze the changes in the gut microbiota following the drug treatment. The fecal samples were stored at −80°C on day 0, day 4, day 6, day 10, day 18, and day 26 for subsequent 16S rDNA analysis. Genomic DNA from the ground fecal samples was extracted using the Fecal Genomic DNA Extraction Kit (Beijing, Solarbio) for library construction and analysis. Amplification was performed using the primers 515F (5′-GTGCCAGCMGCCGCGGTAA-3′) and 907R (5′-CCG​TCA​ATT​CCT​TTG​AGT​TT-3′). Microbiome bioinformatics analysis was performed using QIIME 2 2019.4 (Bolyen et al., 2019). The sequences were subjected to quality filtering, denoising, merging, and removal of chimeras using the DADA2 plugin (Callahan et al., 2016). The amplicon sequence variants (ASVs) were classified using the classifier (classify-sklearn naive Bayes) from the Feature Classifier plugin, based on the Greengenes 13_8 99% OTU reference sequences (McDonald et al., 2012).

### 2.15 Pharmacokinetics of halicin

Eight SD rats weighing 200 g on average were used. They were split into two groups, each of which had two males and two females. The drugs were dissolved in a 0.5% CMC-Na aqueous solution to achieve the desired concentration and mixed thoroughly. The medication was given as a single oral gavage at a dose of 1 mL per 100 g body weight. A high-dose group (50 mg/kg) and a low-dose group (10 mg/kg) were set. Blood samples were taken at 0, 1, 2, 4, 8, 12, and 24 h after administration. Liquid chromatography-mass spectrometry (PerkinElmer, Qsight 210) analysis was conducted for the determination of drug levels in the plasma (Q1 262, Q3 133). The 0.1% formic acid in water and acetonitrile were used as the mobile phase. The time-plasma concentration data in the plasma were analyzed and calculated using the PK Solver plugin in Excel, and noncompartmental modeling was employed to calculate various PK parameters.

### 2.16 Mouse intestinal infection model

We prepared an *in vivo* infection model using 24 BALB/c mice (*n* = 6 per group) weighing 18–22 g. A single colony of *Clostridium perfringens* Type A was inoculated into 10 mL of liver broth gastric digestion medium. The culture was grown in an anaerobic chamber for 16 h, and the pellet was resuspended in PBS buffer. Bacterial counts were determined using the agar plate count method. Based on preliminary experiments, the mouse infection model was established through oral administration (10^9^ CFU/mL). One hour after bacterial challenge, halicin was injected intraperitoneally. The mice were divided into four groups: the high-dose group (10 mg/kg), the low-dose group (5 mg/kg), the negative control group (0 mg/kg), and the positive control group (metronidazole, 10 mg/kg). The survival state of mice was observed and recorded.

### 2.17 Statistical analysis

The LD_50_ of halicin was calculated using the Karber formula and visualized using GraphPad Prism 8.0.2. The data for daily weight gain, food consumption, and organ weights were presented as mean ± standard deviation. Analysis was performed using one-way analysis of variance (ANOVA) to determine significant differences. Pairwise comparisons between all groups and the control group were conducted using the t-test. Significance was reported for results with a *p* < 0.05.

## 3 Results and discussion

### 3.1 Evaluation of antibacterial activity of halicin *in vitro*

As shown in Table 1, the *in vitro* activity test results demonstrated that halicin exhibited broad-spectrum antibacterial activity. Except for *Bacillus subtilis* and *Pseudomonas aeruginosa*, the MIC of halicin against other bacteria was less than 8 μg/mL.

**TABLE 1:** Antimicrobial activities of halicin (MIC, μg/mL).

Strain and number (G^−^)	MIC (μg/mL)	Strain and number (G^+^)	MIC (μg/mL)
*E. coli* (ATCC 25922)	8	*S. aureus* (ATCC 29213)	8
*Salmonella* (CVCC 3377)	8	*E. faecalis* (ATCC 29212)	4
*Proteus* (CMCC (B) 49027)	8	*B. subtilis* (ATCC 66333)	128
*K. pneumoniae* (ATCC 4352)	8	MRSA (ATCC 43300)	8
*S. flexneri* (CMCC 51572)	8	MRSA (BNCC 337371)	8
*R. anatipestifer* (ATCC 11845)	4	*S. pneumoniae* (ATCC 49619)	0.5
*P. aeruginosa* (ATCC 27853)	64	*C. perfringens* (ATCC 13124)	8
*A. baumannii* (ATCC 19606)	8	*Streptococcus suis* (ATCC 43765)	4
*H. parasuis* (ATCC 19417)	8		
*R. anatipestife* (ATCC 11845)	8		

^a^ MRSA, Methicillin resistant *S*. *aureus*.

**FIGURE 1:**
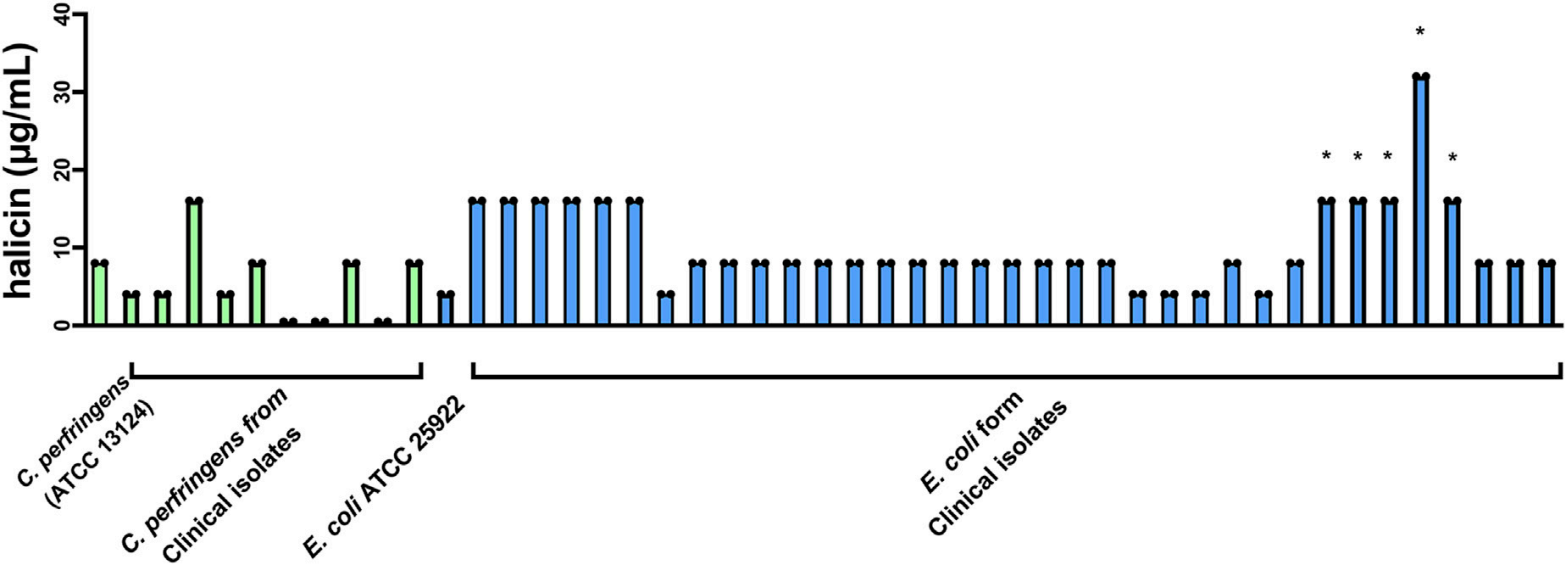
Determination of the MIC of halicin against clinical isolates of *C. perfringens* (green) and *E. coli* (blue). *: sulfonamide-resistant *E. coli*. Strain number and MIC are listed in Supplementary Table S1.

At the same time, we found that halicin had a good inhibitory effect (MIC, 8 μg/mL) on *C. perfringens*. We conducted *in vitro* antimicrobial experiments using 10 clinical isolates of *C. perfringens* strains and 36 clinical isolates of *E. coli* strains. The results showed that the MICs of halicin ranged from 0.5 to 16 μg/mL against clinical isolates of *C. perfringens* strains, and from 4 to 16 μg/mL against clinical isolates of *E. coli* strains (Figure 1). This indicates that halicin has a strong antibacterial activity against both bacteria. Interestingly, we found that *E. coli* with sulfonamide resistance genes showed an increase in the minimum inhibitory concentration (MIC, 16–32 μg/mL) when faced with halicin. This may imply that the mechanism of action of halicin is similar to that of sulfonamide drugs, and that sulfonamide resistance genes affect the sensitivity of resistant strains to halicin.

### 3.2 Resistance development and growth kinetics curve

**FIGURE 2:**
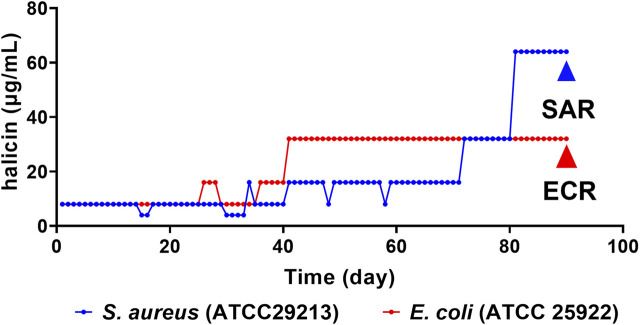
Multipassage resistance studies of halicin against *S. aureus* (ATCC 29213, blue) and *E. coli* (ATCC 25922, red). SAR (*S. aureus* ATCC 29213 resistant strains), MIC = 64 μg/mL; ECR (*E. coli* ATCC 25922 resistant strains), MIC = 32 μg/mL.

After 90 days of continuous exposure to subminimal inhibitory concentration (0.5 × MIC) of halicin (Figure 2), the MIC of *S. aureus* (ATCC 29213) increased from 8 to 64 μg/mL. Resistant strains were isolated and sequenced using a second-generation sequencing scheme to identify the main cause of the increase in MIC. No clear genomic differences were observed between susceptible *S. aureus* (ATCC 29213, MIC 8 μg/mL) and resistant *S. aureus* strains (SAR, MIC 64 μg/mL), indicating that halicin resistance in SAR may be mediated by small chromosomal changes. Compared with susceptible *S. aureus* (ATCC 29213), SAR revealed 18 SNPs in the coding regions (Table 2). SNP analysis of SAR suggested that resistance to halcin may be related to bacterial protein synthesis (*priA*, *prmA* and *rot*), transport (*metC and agcS*), methylation regulation (*miaB*) and nitroreduction (*nadE*). *nadE* mutations are important for metronidazole resistance (Danecek et al., 2011). The conversion of cytosine to thymine at nucleotide position 1,955,821 results in the conversion of arginine (codon CGA) to glutaminic acid (codon CAA) at amino acid position 188 of *the nadE* protein.

**TABLE 2:** Non-synonymous mutations identified in *S. aureus* resistant strains compared with *S. aureus*.

Gene ID	Position	References	Nucleotide variation	AA change	Gene	Annotation
NZ_CP094857.1	89038	C	G	P227A		Glycosyltransferase family 4 protein
NZ_CP094857.1	349779	C	T	V317M	*metC*	Bifunctional cystathionine gamma-lyase/gamma-synthase
NZ_CP094857.1	625195	G	C	E510D		Cation/H^+^ exchanger domain-containing protein
NZ_CP094857.1	770810	G	A	R275P		Nucleotide-binding protein NWMN_0733
NZ_CP094857.1	912499	C	G	P20G		Putative undecaprenyl phosphate transporter
NZ_CP094857.1	1176345	C	T	A365V	*priA*	Primosomal protein N′
NZ_CP094857.1	1270992	G	C	G266A	*miaB*	tRNA-2-methylthio-N (6)-dimethylallyladenosine synthase
NZ_CP094857.1	1279253	G	A	G222S		Aquaporin family protein
NZ_CP094857.1	1340970	G	C	G71R	*agcS*	Amino acid carrier protein
NZ_CP094857.1	1396378	G	A	P231L		VWFA domain-containing protein
NZ_CP094857.1	1570973	C	T	E144K	*pbp3*	Penicillin-binding protein 3
NZ_CP094857.1	1595593	C	T	E33K	*prmA*	Ribosomal protein L11 methyltransferase
NZ_CP094857.1	1646174	G	A	T336I	*hisS*	Histidine--tRNA ligase
NZ_CP094857.1	1798638	C	T	R68H	*rot*	HTH-type transcriptional regulator rot
NZ_CP094857.1	1955821	C	T	R189Q	*nadE*	NH(3)-dependent NAD (+) synthetase
NZ_CP094857.1	2058829	G	A	G196R	*ilvD*	Dihydroxy-acid dehydratase
NZ_CP094857.1	2190609	C	T	L301F		Major facilitator superfamily (MFS) profile
NZ_CP094857.1	2550501	G	C	R88A		Putative NAD(P)H nitroreductase

The meaning of the italic values represents the name of the gene encoding the protein.

The main active group of metronidazole is 5-nitro group, and its antibacterial activity largely depends on the reduction reaction (Oliveira et al., 2018). The *nadE* gene encodes the NAD⁺ synthase, a crucial enzyme in NAD⁺ biosynthesis that is essential for maintaining intracellular NAD⁺ levels. NAD⁺ is a vital cofactor involved in various biochemical reactions, and affects the cellular reduction environment (Santos et al., 2020). Mutation or expression changes in the *nadE* gene can lead to adjustments in NAD⁺ levels, which alters the state of cellular reduction, affects the activity of metronidazole and increases bacterial resistance to metronidazole. Given the structural similarity between the nitro group of halicin and metronidazole, we speculate that this is also the main reason for SAR resistance to halicin. Therefore, before halicin is applied in the clinical treatment of infections with multidrug-resistant pathogens, investigating the effect of altered NAD⁺ levels on halicin activity and how variations in the *nadE* gene contribute to the formation of resistance is a key step in developing effective treatment protocols and preventing the development of resistance.

**FIGURE 3:**
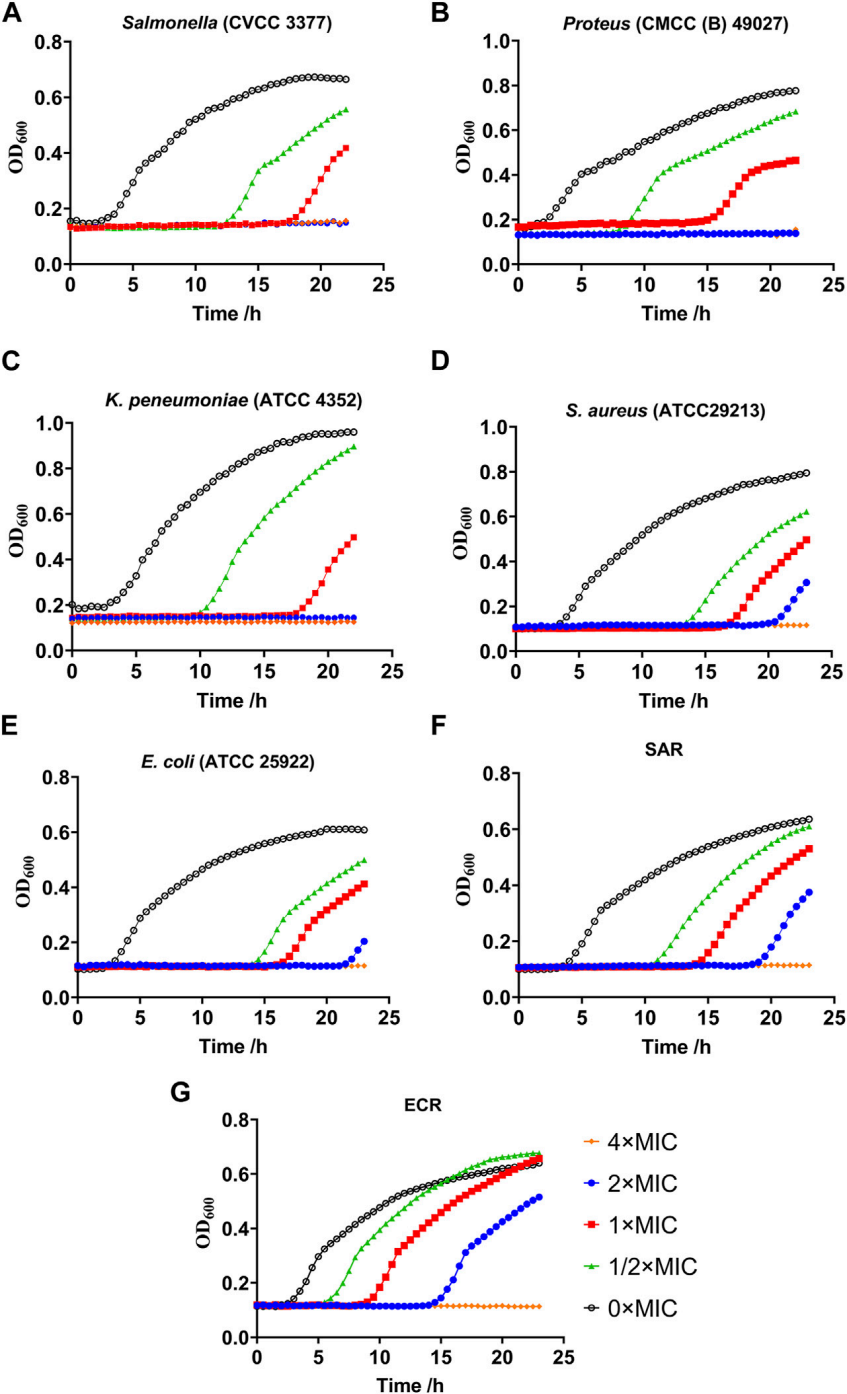
Growth kinetics curve assays of resistant *Salmonella* **(A)**, *Proteus* **(B)**, *Klebsiella pneumoniae* **(C)**, *S. aureus* **(D)**, *E. coli* **(E)**, SAR (*S. aureus* ATCC 29213 resistant strains) **(F)** and ECR (*E. coli* ATCC 25922 resistant strains) **(G)**.

Growth kinetics curve assays showed that *Salmonella*, *Proteus* and *K. pneumoniae* were completely inhibited by halicin at 1/2 × to 4 × MIC (Figures 3A–C). Interestingly, *S. aureus* and *E. coli* still proliferated significantly *in vitro* under halicin treatment at 4 × MIC after 20 h. We compared the growth rates of the induced resistant bacteria (SAR and ECR) and the wild-type strains *S. aureus* (ATCC 29313) and *E. coli* (ATCC 25922) (Figures 3D–G). A specific phenomenon was observed where halicin-treated *S. aureus* started to grow rapidly after 16 h at 1 × MIC. This result confirmed that halicin was a bacteriostatic rather than a bactericidal agent against *S. aureus*. A similar phenomenon was observed in *E. coli* (ATCC 25922) and its resistant strain ECR. However, ECR exhibited a higher bacterial growth rate. At 1/2 × MIC, the growth of *E. coli* (ATCC 25922) was significantly inhibited during the initial 16 h. However, ECR exhibited noticeable growth at the 8 h.

### 3.3 Toxicity evaluation of halicin

#### 3.3.1 Ames test

The Ames test is a biological assay that assesses the mutagenic potential of chemical compounds by exposing amino acid-requiring organisms to varying concentrations of chemicals and selecting for a reversion event. Only those cells that have undergone reversion to histidine/tryptophan prototrophy survive and grow (Guy, 2024). The halicin induced mutations in *Salmonella typhimurium* with or without S9 metabolic activation are shown in Table 3. The bacterial counts of all positive groups were more than twice that of the solvent control group. Halicin exhibited non-mutagenic characteristics at concentrations below 11.2 μg/plate. However, at 22.5 μg/plate of halicin, the bacterial counts of the four strains significantly decreased due to its antibacterial effect. At 11.2 μg/plate, halicin also significantly reduced the bacterial counts of TA100 (-S9 and +S9). Therefore, the Ames test cannot be used to determine the mutagenic effects of halicin at higher concentrations. This result may be attributed to halicin’s bacteriostatic ability.

**TABLE 3:** Ames test results of halicin using *Salmonella typhimurium* strains TA97, TA98, TA100, and WP2uvrApKM101.

Treatment	Does (μg/plate)	TA97a		TA98		TA100		WP2uvApKM101	
		+S9	-S9	+S9	-S9	+S9	-S9	+S9	-S9
Vehicle control	0	117 ± 2.8	84 ± 5.7	31 ± 5.7	32 ± 2.8	140 ± 12.7	82 ± 4.2	181 ± 11.3	131 ± 7.1
2-AF	10	2052 ± 91.9	-	3,219 ± 106.1	-	2,234 ± 29.7	-	-	-
DDT	50	-	3,451 ± 53.7	-	2,985 ± 76.4	-	-	-	-
MSM	1	-	-	-	-	-	3,042 ± 69.3	-	926 ± 45.3
2-AT	10	-	-	-	-	-	-	546 ± 26.9	-
Halicin	45	0 ± 0	0 ± 0	0 ± 0	0 ± 0	0 ± 0	0 ± 0	0 ± 0	0 ± 0
	22.5	31 ± 8.5	0 ± 0	53 ± 7.1	55 ± 5.7	0 ± 0	0 ± 0	12 ± 5.7	75 ± 8.5
	11.2	151 ± 11.3	115 ± 8.5	43 ± 8.5	61 ± 14.1	152 ± 12.7	0 ± 0	168 ± 19.8	135 ± 22.6
	5.6	145 ± 5.7	82 ± 14.1	30 ± 12.7	27 ± 2.8	171 ± 11.3	121 ± 9.9	183 ± 15.6	169 ± 15.6

^a^ 2-AF: 2-Aminofluorene; DDT: fenaminosulf; MSM: methyl methanesulfonate; 2-AT: 2-Aminoanthracene.

#### 3.3.2 Acute oral toxicity test in ICR mice

**FIGURE 4:**
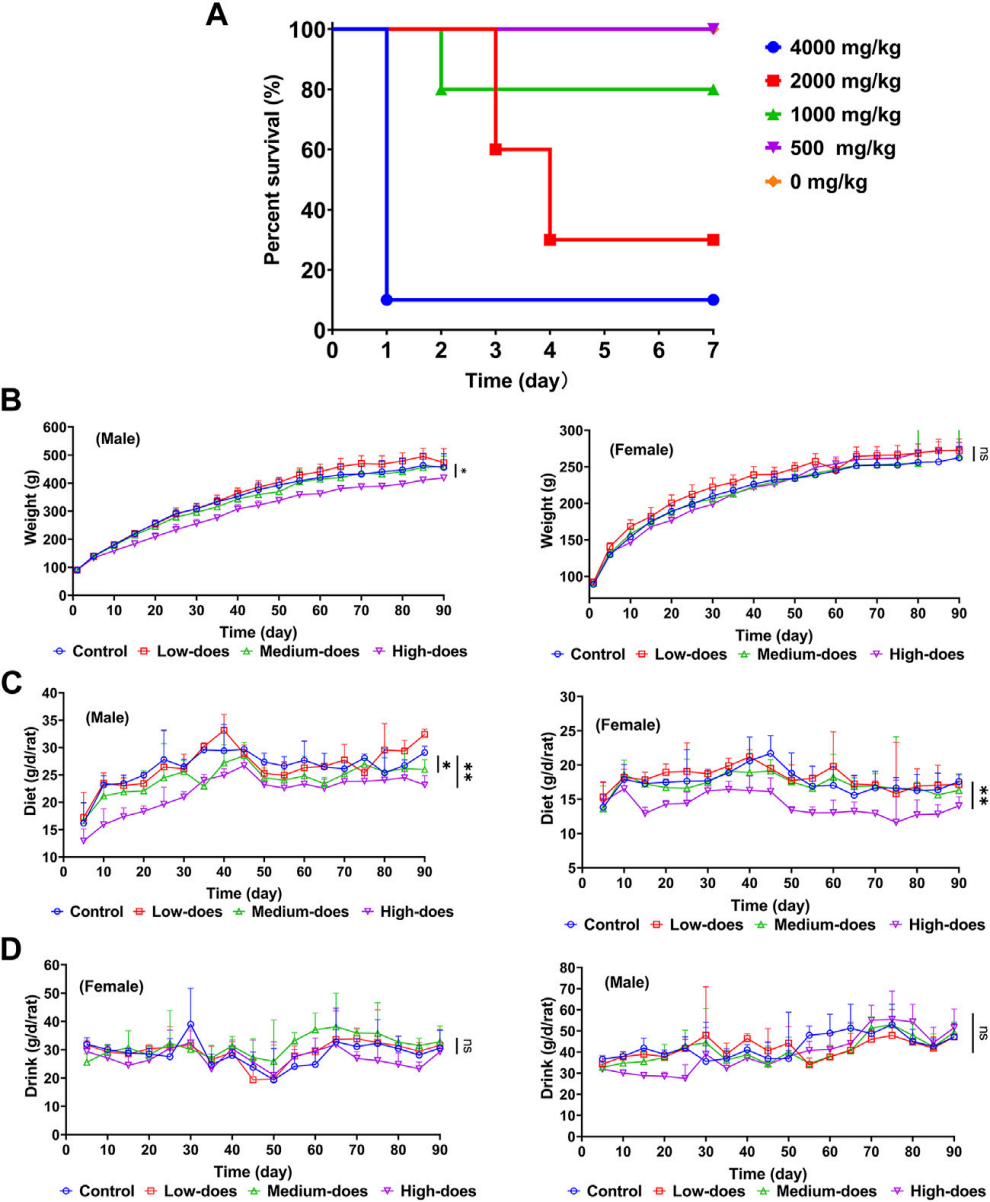
Safety evaluation of acute and subchronic toxicity of halicin. **(A)** Acute oral toxicity test of halicin in mice, LD_50_ = 2018.3 mg/kg (95% confidence interval: 1510.0 mg/kg-2738.3 mg/kg). **(B–D)**: 90-day subchronic toxicity test of halicin, assessing changes in rat body weight **(B)**, diet **(C)** and drinking **(D)**. Each dose group contains 5 female and 5 male rats (*, *p* < 0.05; **, *p* < 0.01; ns, *p* > 0.05). High-dose, 201.8 mg/kg; medium-dose, 100.9 mg/kg; low-dose, 50.5 mg/kg.

We studied the acute oral toxicity of halicin *in vivo*, as this could provide a dose reference for further toxicity evaluation (Figure 4A). At a dose of 4,000 mg/kg, ICR mice showed clinical symptoms of poisoning such as reduced motor activity, weakness, and drowsiness, and died within 2–24 h with a 7-day survival rate of 10%. At a dose of 2000 mg/kg, the mice died 3–4 days after administration. At a dose of 1,000 mg/kg, only a few mice died on the second day and the survival rate was 80% at 7 days. At a dose of 500 mg/kg, all mice were normal and none died. Based on the dose-mortality relationship, the LD_50_ of halicin in mice was estimated to be 2018.3 mg/kg (95% confidence interval: 1510.0 mg/kg–2738.3 mg/kg) by the Bliss method.

#### 3.3.3 Mice sperm abnormality test

To further assess the genetic toxicity of halicin, we performed a sperm teratogenicity test in mice. The ratio of sperm morphological abnormalities and the proportions of different types of malformations are shown in Table 4 and Supplementary Figure S1A. The proportions of abnormal sperm in halicin treatment groups (high-dose 1009.1 mg/kg, medium-dose 504.6 mg/kg, and low-dose 252.3 mg/kg) were not significantly different from those in the negative control group (*p* > 0.05), and the ratio of sperm abnormalities in the three groups were significantly lower than in the cyclophosphamide group (positive control), indicating that halicin did not cause sperm morphological abnormalities at the three dose levels.

**TABLE 4:** Sperm abnormality test of halicin in mice.

Parameters	Group (mg/kg)				
	High-dose (1009.1)	Medium-dose (504.6)	Low-dose (252.3)	Negative	Positive
Number of mice	5	5	5	5	5
Number of sperms observed	5 × 1,000	5 × 1,000	5 × 1,000	5 × 1,000	5 × 1,000
Number of sperm abnormality	110	84	78	83	417
Abnormal ratio (%)	2.12 ± 0.38	1.68 ± 0.13	1.56 ± 0.09	1.62 ± 0.15	8.34 ± 0.47
Significance of difference	*p* > 0.05	*p* > 0.05	*p* > 0.05	-	*p* < 0.05
Abnormal sperms counted ratio (%)					
No hook	15.6 ± 5.5	16.7 ± 2.71	16.61 ± 3.11	17.26 ± 4.68	10.07 ± 1.23
Banana shape	42.52 ± 12.57	35.81 ± 4.52	35.98 ± 3.94	28.34 ± 4.12	10.58 ± 1.46
Amorphous	23.6 ± 12.44	29.69 ± 2.67	39.81 ± 3	39.72 ± 4.3	48.33 ± 4.33
Large round head	9.07 ± 4.02	11.96 ± 0.96	3.92 ± 3.58	6.28 ± 4.19	9.37 ± 1.03
Kinks tail	3.05 ± 2.79	2.44 ± 3.37	1.18 ± 2.63	3.61 ± 3.31	5.76 ± 0.94
Two head	3.05 ± 2.79	2.22 ± 3.04	1.33 ± 2.98	2.36 ± 3.24	5.52 ± 1.36
Two tail	3.11 ± 2.84	1.18 ± 2.63	1.18 ± 2.63	2.43 ± 3.33	4.84 ± 1.99

#### 3.3.4 Chromosome aberration test of mammalian bone marrow cells

We further conducted the chromosomal aberration experiment in mouse bone marrow cells (Table 5, Supplementary Figure S1B). There was no significant difference between the negative control group and halicin groups (252.3 mg/kg to 1009.1 mg/kg) (*p* > 0.05). These results indicate that halicin had no significant teratogenic effect on bone marrow cells.

**TABLE 5:** Summary of chromosomal aberration frequencies in mouse bone marrow cells after halicin administration.

Group (mg/kg)	Number of mice	Number of cells at metaphase	Number of cells with chromosome aberration	Chromosome aberration (%)	*P*
High-does (1009.1)	5	5 × 100	7	1.4 ± 0.55	*p* > 0.05
Medium-does (504.6)	5	5 × 100	8	1.6 ± 0.89	*p* > 0.05
Low-does (252.3)	5	5 × 100	7	1.4 ± 1.14	*p* > 0.05
Negative	5	5 × 100	4	0.8 ± 0.84	-
Positive	5	5 × 100	93	18.6 ± 7.67	*p* < 0.05

#### 3.3.5 Micronucleus experiment of mammalian bone marrow cells

To further investigate the impact of halicin on the internal structure of chromosomes, and to ensure the reliability of safety experiments, we conducted a bone marrow erythrocyte micronucleus test in addition to the chromosomal aberration assay. The results showed that there was no significant difference in micronucleus ratio between the negative control group and the medium (504.6 mg/kg) and low-dose (252.3 mg/kg) of halicin groups (*p* > 0.05) (Table 6). However, a significant difference was observed between the high-dose (1009.1 mg/kg) and negative control groups (*p* < 0.01). This suggests that halicin, at a concentration of 1009.1 mg/kg, could lead to cellular stress or toxicological effects. The number of micronuclei in all halicin-treated groups and the negative control group were significantly lower than that in the cyclophosphamide group (*p* < 0.01).

**TABLE 6:** Effects of halicin on bone marrow micronucleus and Polychromatic Erythrocytes/Red Blood Cells (PCE/RBC) ratio in mice.

Sex	Group (mg/kg)	PCE/RBC	PCE micronucleus (‰)	*P*
Female	High-does (1009.1)	0.72 ± 0.08	2.2 ± 0.8	*p* < 0.01
	Medium-does (504.6)	1.06 ± 0.07	0.8 ± 0.4	*p* > 0.05
	Low-does (252.3)	1.09 ± 0.06	1.2 ± 0.4	*p* > 0.05
	Negative	1 ± 0.06	0.6 ± 0.5	-
	Positive	0.5 ± 0.04	18.8 ± 1.9	*p* < 0.01
Male	High-does (1009.1)	0.76 ± 0.03	0.8 ± 0.8	*p* < 0.01
	Medium-does (504.6)	1.08 ± 0.11	1 ± 0.7	*p* > 0.05
	Low-does (252.3)	1.07 ± 0.09	0.6 ± 0.5	*p* > 0.05
	Negative	1 ± 0.05	0.8 ± 0.4	-
	Positive	0.51 ± 0.09	18.8 ± 2.6	*p* < 0.01

Drug toxicity was a crucial factor in determining the viability of a compound as a potential drug candidate. Our study evaluated the toxicity of halicin through various tests, including Ames test, acute oral toxicity, and sperm malformation tests in mice. The results showed that the LD_50_ of halicin was 2018.3 mg/kg, classifying it as a low-toxicity compound (OECD, 2002). Genotoxicity studies showed that halicin did not show significant sperm malformation or chromosomal teratogen damage at doses ranging from 252.3 mg/kg to 1009.1 mg/kg.

#### 3.3.6 90-Day subchronic toxicity assay

**FIGURE 5:**
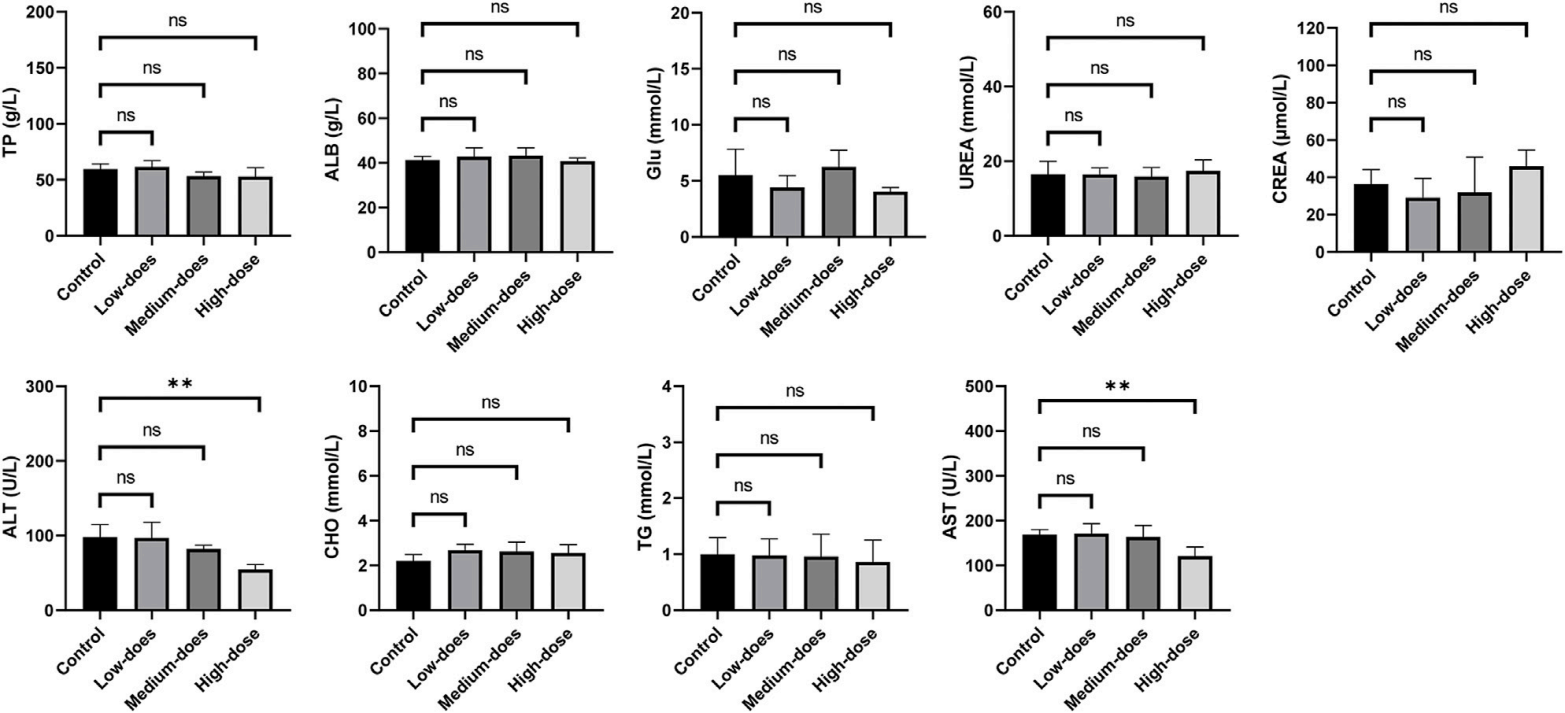
Serum biochemical indicators of 90-day subchronic toxicity test. TP: Total protein; ALB: Albumin; Glu: Glucose; UREA: Urea; CREA: Creatinine; ALT: Alanine aminotransferase; CHO: Cholesterol; TG: Triglycerides; AST: Aspartate aminotransferase. High-dose, 201.8 mg/kg; medium-dose, 100.9 mg/kg; low-dose, 50.5 mg/kg. **, *p* < 0.01.

**FIGURE 6:**
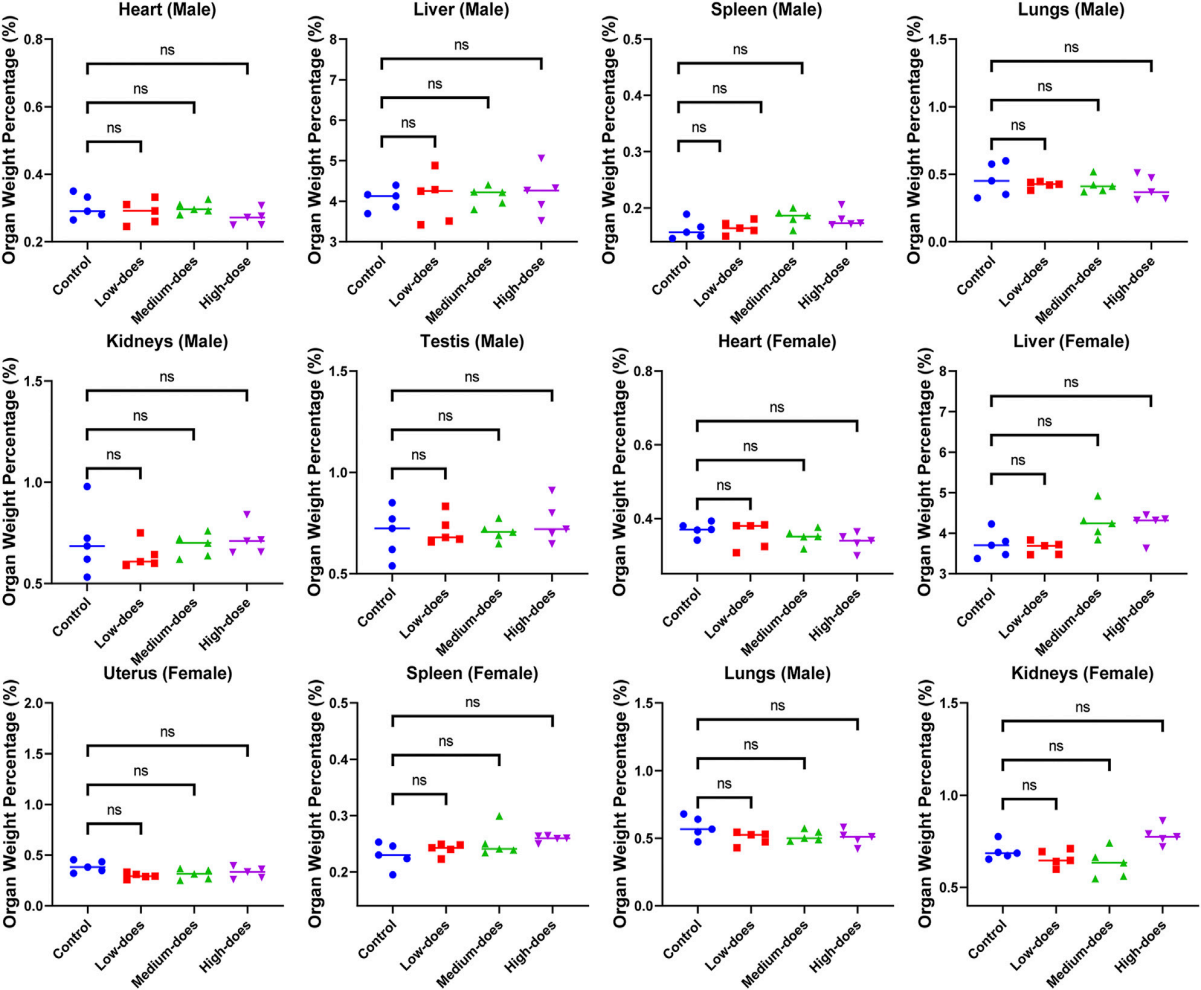
Effect of halicin on organ weight ratio of rats at the end of 90-day subchronic toxicity test. High-dose, 201.8 mg/kg; medium-dose, 100.9 mg/kg; low-dose, 50.5 mg/kg.

**FIGURE 7:**
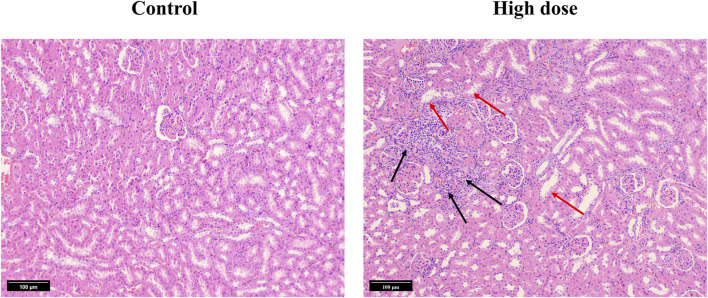
Hematoxylin and Eosin (HE) stained section of kidney tissue from the 90-day subchronic toxicity test of halicin. High-dose, 201.8 mg/kg. The black arrow indicates infiltration of inflammatory cells. The red arrow indicates separation of the basement membrane and epithelial cells.

We subsequently conducted a 90-day subchronic toxicity test to evaluate the long-term effects of halicin on rats. During the experiment, we monitored the body weight, diet, and water intake of the model animals. The results showed that there was no significant difference in the body weight of female rats between the control group and halicin treatment groups during the experimental period (*p* > 0.05). However, the body weight of male rats in the high-dose group (201.8 mg/kg) was significantly lower than that of the control group (*p* < 0.05) (Figure 4B). This might be related to the different degree of drug metabolism in their bodies. In terms of diet, there were significant differences between the control and the high and medium dose of halicin treatment groups of male rats. No significant differences were noted between the control group and the low-dose group (50.5 mg/kg) (*p* > 0.05) during the 90-day diet monitoring. In female rats, only the high-dose group (201.8 mg/kg) was significantly different from the control group (*p* < 0.01) (Figure 4C). In addition, it was observed that there were no significant changes in water consumption and mental states across all dosage groups (Figure 4D). No mortality was recorded in any experimental group. Moreover, we examined a large number of blood indicators at 45 and 90 days, most of which did not show significant differences (Figure 5; Supplementary Figure S2–S4). It was found that high-dose halicin treatment significantly reduced the levels of serum alanine aminotransferase (ALT) and aspartate aminotransferase (AST) in rats compared with the control group (*p* < 0.01). The emergence of these results may be related to the renal toxicity caused by halicin. We checked the reference ranges for the hematology and serum biochemistry indices provided by the manufacturer of the purchased rats and found that their values were within the normal range (Kunutsor et al., 2015; Askari et al., 2016). At the end of the experiment, the rats were autopsied and the key organs were weighed (Figure 6). The results showed that there was no significant difference in the weight of organs between the control group and the halicin treatment groups (*p* > 0.05). During autopsy, spotty lesions were found in the kidneys of the high-dose group, and glomerulopathy was present. HE sections of kidney tissue showed that high dose of halicin (201.8 mg/kg) caused kidney lesions in rats, including separation of basement membrane and epithelial cells, and a small amount of inflammatory cell infiltration, which indicated that high dose of halicin had certain renal toxicity (Figure 7).

These findings provide important insight that halicin has a good safety and tolerability at dosages below 50.5 mg/kg, justifying further investigation into its potential therapeutic applications. It is important to note, however, that further research is necessary to fully understand the safety and efficacy of halicin in different animal models and ultimately in human subjects. Nonetheless, these results provide insight into the potential of halicin as a therapeutic agent (Dong et al., 2022a).

#### 3.3.7 Acute embryotoxicity and embryonic malformation studies in zebrafish

**FIGURE 8:**
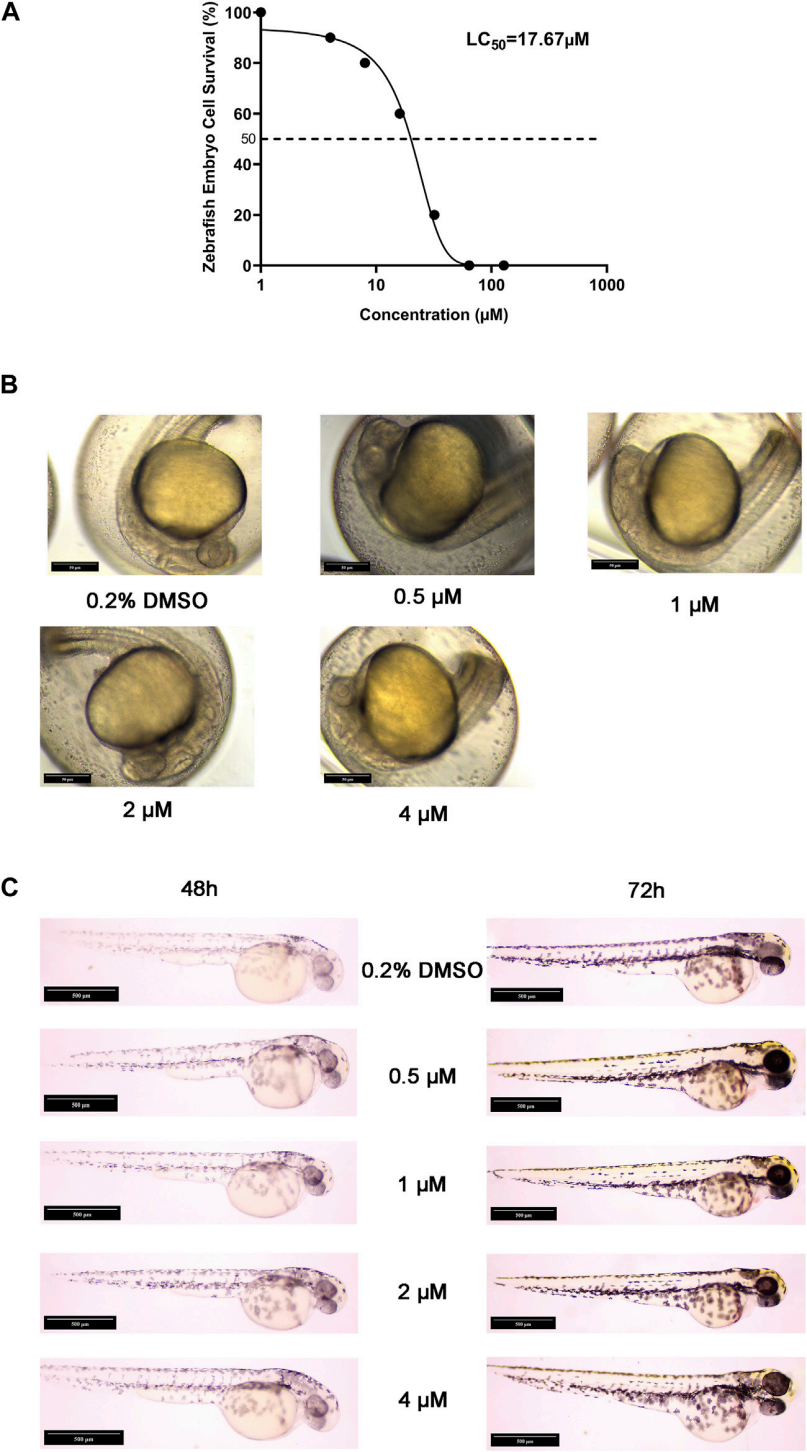
Acute embryotoxicity and embryonic malformation studies in zebrafish. **(A)** The LC_50_ value of halicin was determined with a 95% confidence interval ranging from 13.88 to 22.12 μM. **(B)** Zebrafish embryo sections at 24 h after fertilization. **(C)** Zebrafish embryo sections at 48 h (Left) and 72 h (Right) after fertilization.

Zebrafish were an excellent model organism, especially for the study of drug-induced embryonic teratogenesis, as they allowed for more direct and clear observation of embryonic conditions (Song et al., 2021). To investigate the genotoxicity of halicin, we utilized zebrafish for an embryonic teratogenicity experiment. Our findings indicate that the LC_50_ of halicin in zebrafish embryos is 17.64 μM (Figure 8A). We selected 4 μM as the maximum concentration for teratogenicity studies and observed the yolk sac, heart, body shape, tail, head, and ears of the zebrafish embryos at 24, 48, and 72 h after fertilization (Figures 8B, C). The results demonstrated no any significant teratogenic effects of halicin on the embryos, which is a cause for celebration.

### 3.4 Microbiological analysis

**FIGURE 9:**
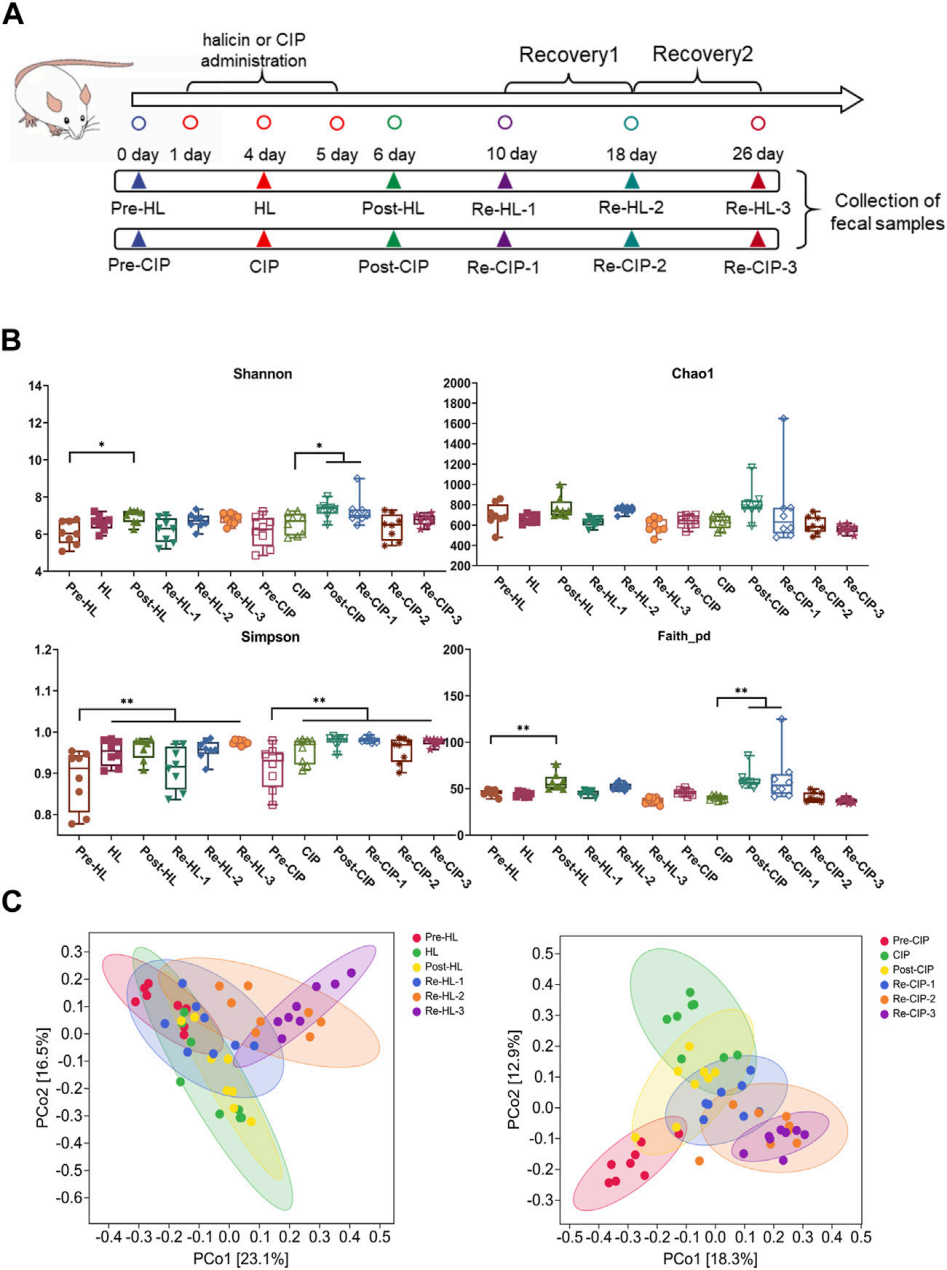
Changes in the richness and diversity of the fecal microbiota community of mice after antibiotic treatment. BALB/c mice (*n* = 10) were treated with halicin and ciprofloxacin for 3 days, respectively, and monitored for recovery after antibiotic withdrawal. **(A)** Experimental grouping and sampling arrangement. The pre-HL represents fecal samples from mice before halicin treatment; HL represents fecal samples from the first day after treatment; Post-HL represents fecal samples from the last day of halicin treatment; RE-HL represents fecal samples after halicin withdrawal; pre-CIP represents fecal samples from mice before ciprofloxacin treatment; CIP represents fecal samples from the first day after treatment; Post-CIP represents fecal samples from the last day of ciprofloxacin treatment; RE-CIP represents fecal samples after ciprofloxacin withdrawal. **(B)** Bacterial α-diversity analysis of fecal samples from different treatment groups. **(C)** β-diversity analysis was performed by PcoA methods.

The *in vitro* activity and toxicity data suggest that halicin is suitable as a potential antibiotic for the treatment of intestinal bacterial infections. However, the effect of halicin on the intestinal microbiota has not been reported. Therefore, we investigated the effects of 15 mg/kg halicin to conduct intestinal microbiota (Figure 9A). The richness and diversity of the microbial community are reflected in its alpha diversity. Ciprofloxacin (CIP), a commonly used drug for the treatment of intestinal bacterial infections, was used as a positive control. We found that halicin and CIP increased Shannon (*p* < 0.05) and Faith_pd levels (*p* < 0.01) 1 day after withdrawal. The results indicated that the diversity and richness of the intestinal microbiota increased significantly after withdrawal, which may be because halicin and CIP are broad-spectrum antibiotics, which have a great impact on the intestinal microbiota after administration, resulting in intestinal microbiota disorder. In the halicin group, the Shannon and Faith_pd values returned to normal levels in the first recovery period, whereas in the CIP group, the Shannon and Faith_pd values recovered in the second recovery period (Figure 9B). We used PCoA methods to analyze beta diversity (Figure 9C). Halicin-treated samples showed no significant separation as the experiment progressed, whereas CIP-treated samples showed significant changes in microbial community. These results indicated that although halicin had a significant effect on gut microbiota, it made them recover faster. CIP treatment caused significant changes in the structure of gut microbiota, which did not return to normal levels 21 days after withdrawal.

**FIGURE 10:**
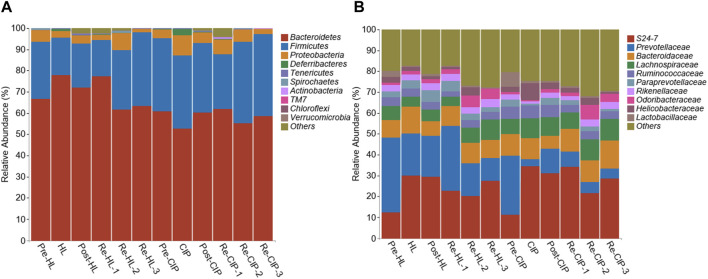
Composition of the fecal microbiota of mice before and after halicin or CIP treatment at the phylum **(A)** and genus **(B)** level.

The relative proportions of the different taxa were evaluated at the phylum, class, and genus levels (Figure 10). At the phylum level, there was no clear change before and after administration, and phyla mainly included Bacteroidetes, Firmicutes and Proteobacteria, accounting for 94.7%–99.59% of the total proportion, among which Bacteroidetes had the highest proportion (Figure 10A). At the genus level, the main taxon was *S24-7*, *Prevotellaceae*, *Bacteroidaceae*, *Lachnospiraceae*, *Ruminococcaceae*, *Paraprevotellaceae*, *Rikenellaceae*, *Odoribacteraceae*, *Helicobacteraceae*, and *Lactobacillaceae*. Halicin and CIP caused a significant decrease in the content of *Prevotellaceae* and *Lactobacillaceae* after administration. Halicin had less effect on the *Prevotellaceae* and *Lactobacillaceae* abundance than CIP, which did not restore these two genera to normal levels during the third recovery period (Figure 10B).

**FIGURE 11:**
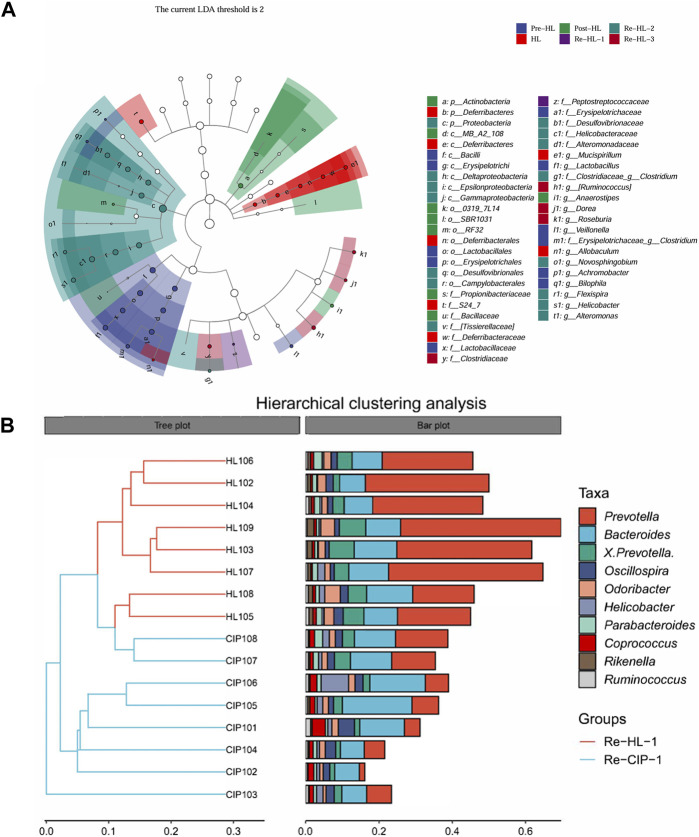
Differentially represented bacterial families after antibiotic treatment with halicin or CIP compared to no treatment. **(A)** Lefse analysis identified the microbes that showed significant differences in gut microbiota abundance at different time points of halicin treatment. **(B)** Differences in intestinal flora between the HL and CIP groups on the first day of the recovery period.

To further explore the effect of halicin on the intestinal bacterial structure at different time points, we performed Lefse analysis to identify different species at different time points (Figure 11A; Supplementary Figure S5). In the fecal samples collected during administration, there was no significant difference between the CIP-treated and halicin-treated groups at the phylum level; however, we found a special species in each group. In the halicin group, the abundance of *g_Allobaculum*, which has been reported to be associated with beneficial effects in organisms, was increased. In the CIP group administration, we found *g_Mucispirillum*, which has a protective effect on the intestinal mucosa, but mainly attaches to the intestinal epithelial mucosa and is not often found in feces (Supplementary Figure S5). We speculate that this may be related to serious damage to the mouse intestinal mucosal layer after CIP treatment, which affects the colonization of *g_Mucispirillum* in the intestinal mucosal layer. In the halicin group, we identified 15 marker species during the second recovery period, including *p_Proteobacteria, o_Campylobacterales, c_Epsilonproteobacteria, f_Helicobacteraceae, g_Helicobacter, c_Gammaproteobacteria, c_Deltaproteobacteria, f_Desulfovibrionaceae, o_Desulfovibrionales, g_Flexispira, g_Novosphingobium, f_Alteromonadaceae, g_Alteromonas, f_Tissierellaceae, f_Clostridiaceae, g_Clostridium.* Interestingly, most of these marker species belonged to the phylum *Proteobacteria*. In the first recovery period, the CIP group had more marker species than the halicin group, totaling more than 50 specific species, most of which belonged to the phylum *Proteobacteria*. However, among these marker species, we identified many bacteria that have not been previously reported in the intestinal microbiota. We believe that these bacteria appeared because CIP seriously damaged the mouse intestinal microbiota structure, causing it to lose its resistance to external bacteria colonization. We performed cluster analysis on Re-HL-1 and Re-CIP-1 groups, and showed obvious cluster differences between the two groups (Figure 11B). This result showed that CIP severely disrupted the homeostasis of the gut microbial system in mice during this period.

### 3.5 Pharmacokinetics in rat

Further studies are necessary to fully elucidate the mechanism of action and the potential clinical applications of halicin. The pharmacokinetics (PK) of a drug determines its method of use and range of applications. Therefore, we performed a preliminary evaluation of the oral PK of halicin.

**FIGURE 12:**
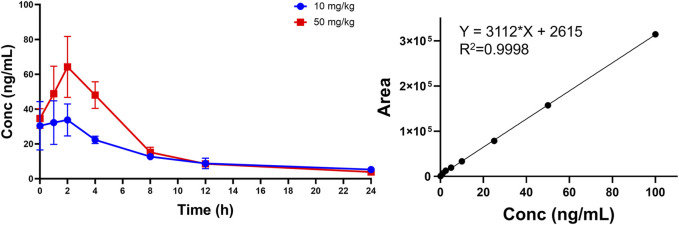
Plasma concentration-time curve of halicin after oral administration.

We used a non-compartmental method (NCA) for PK modeling and PK parameter calculations (Table 7; Figure 12) (Shikov et al., 2020). Halicin had a peak plasma concentration (C_max_) of 64.25 ± 1.98 ng/mL, and a T_1/2_λz between 8–10 h. The drug concentration in the blood was ≤5 ng/mL at 24 h after drug administration. The PK parameters indicated that halicin has a high elimination rate and low blood concentration, making it unsuitable for the treatment of systemic infections. Multiple doses may be required to increase the drug concentration in the blood during treatment. According to the PK parameters and *in vitro* activity experiments, halicin is suitable for intestinal treatment.

**TABLE 7:** PK parameters of halicin in rat.

PK parameter	Low dose (10 mg/kg)	High dose (50 mg/kg)
λz (1/h)	0.068 ± 0.016	0.085 ± 0.0016
T_1/2_λz (h)	10.32 ± 2.22	8.57 ± 0.22
T_max_ (h)	1.33 ± 1.41	1.33 ± 1.41
C_max_ (ng/mL)	40.99 ± 2.37	64.25 ± 1.98
AUC_0-last_ (h·ng/mL)	318.05 ± 31.27	459.58 ± 34.53
AUC_0-∞_ (h·ng/mL)	395.88 ± 42.41	510.86 ± 46.62
Vd/F (mL/kg)	375074.55 ± 41944.37	828269.08 ± 70840.29
Cl/F (mL/h/kg)	25360.4 ± 2661.85	58983.17 ± 7041.30
MRT_last_ (h)	7.58 ± 0.41	5.85 ± 0.13

### 3.6 Effects of halicin on intestinal infection model in mice

*C. perfringens* was an anaerobic, gas-producing, spore-forming bacterium that was commonly found in the intestines of humans and animals (Mehdizadeh Gohari et al., 2021). Due to its ability to produce toxins and cause tissue necrosis, *C. perfringens* infections could lead to severe intestinal diseases. *In vitro* studies, we found that *C. perfringens* was sensitive to halicin. We established a mouse intestinal infection model using Ciprofloxacin-resistant *C. perfringens* isolated from clinical samples via gavage (10^9^ CFU/mL) and evaluated the therapeutic effect of halicin on it.

**FIGURE 13:**
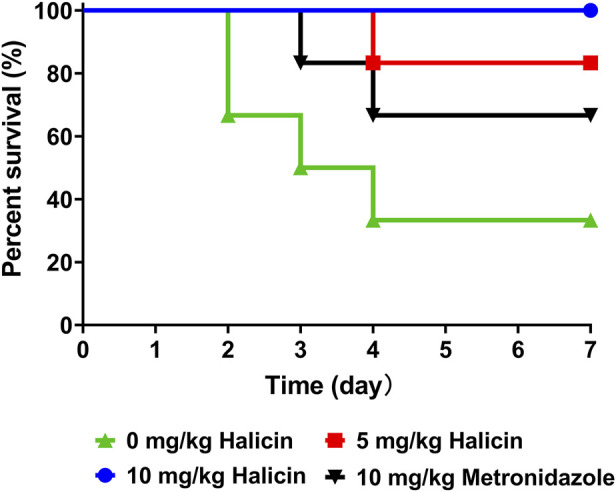
Effects of halicin on a mice model of *C. perfringens* infection.

The mice in the control group (0 mg/kg halicin) had a high mortality rate and appeared wrinkle and abdominal distension 1 day after administration (Figure 13). Within 4 d, 66% of the mice died, and the intestines of the dead mice were inflated. All mice in the 10 mg/kg halicin group survived, and more than 80% of the mice in the 5 mg/kg halicin group survived, and most of the mice did not show ruffled fur. Halicin was much more effective in treating intestinal anaerobic bacteria infections than 10 mg/kg metronidazole. Considering the dosing and LD_50_ doses, we believe that halicin is a potential drug for the treatment of *C. perfringens* infections.

## 4 Conclusion

Halicin, an antibiotic that was discovered using artificial intelligence, exhibits broad-spectrum antibacterial activities and can effectively inhibiting both Gram-positive and Gram-negative bacteria, particularly multidrug-resistant strains. Continuous induction experiments demonstrated that bacteria did not easily develop resistance to halicin. Sequencing of halicin-resistant mutants revealed that the primary mutations were concentrated in three functions: bacterial protein synthesis, transport, and nitroreduction. We found that the *nadE* gene, which mediates resistance to metronidazole, may play a significant role in resistance to halicin. Pharmacokinetic parameters in rats indicated that halicin was rapidly eliminated and had low plasma concentration, rendering it unsuitable for treating systemic infections. Given its antibacterial mechanism and activity spectrum, halicin is better suited as a therapeutic agent for intestinal bacterial infections. Oral acute and subchronic toxicity studies indicated that halicin was generally safe but caused some kidney damage at high doses. Halicin did not significantly affect mouse sperm morphology or bone marrow cell chromosomes. However, high doses of halicin (1009.1 mg/kg) influenced the micronucleus ratio in mouse bone marrow erythrocytes. Halicin significantly affected the mouse gut microbiota, but it demonstrated a rapid recovery of these effects. We validated the therapeutic effect of halicin on mice infected with *C. perfringens*. The results showed that halicin had good therapeutic effects at a dose of 5 mg/kg, and no deaths occurred in the group given a dose of 10 mg/kg. In this study, we determined the maximum safe concentration of halicin to be 50.5 mg/kg through an exhaustive safety assessment. It is worth noting that in our study, the effective concentration clinically used to treat intestinal bacterial infections was significantly lower than this value. These results suggest that halicin has a wide range of safety in clinical application, which provides a basis for further optimization of drug dosage and enhancement of efficacy. In summary, halicin is a suitable candidate drug for the treatment of intestinal infections.

## Data Availability

The datasets presented in this study can be found in online repositories. The names of the repository/repositories and accession number(s) can be found below: https://www.ncbi.nlm.nih.gov/, PRJNA1031798 https://www.ncbi.nlm.nih.gov/, PRJNA1031775.
