# Supplementary material for: Safety and efficacy evaluation of halicin as an effective drug for inhibiting intestinal infections
Source: Front Pharmacol. 2024 May 9;15:1389293. doi: 10.3389/fphar.2024.1389293 (PMC11111955; doi:10.3389/fphar.2024.1389293)
Supplement: Supplementary file 2 [file DataSheet1.docx]

Supplementary Material


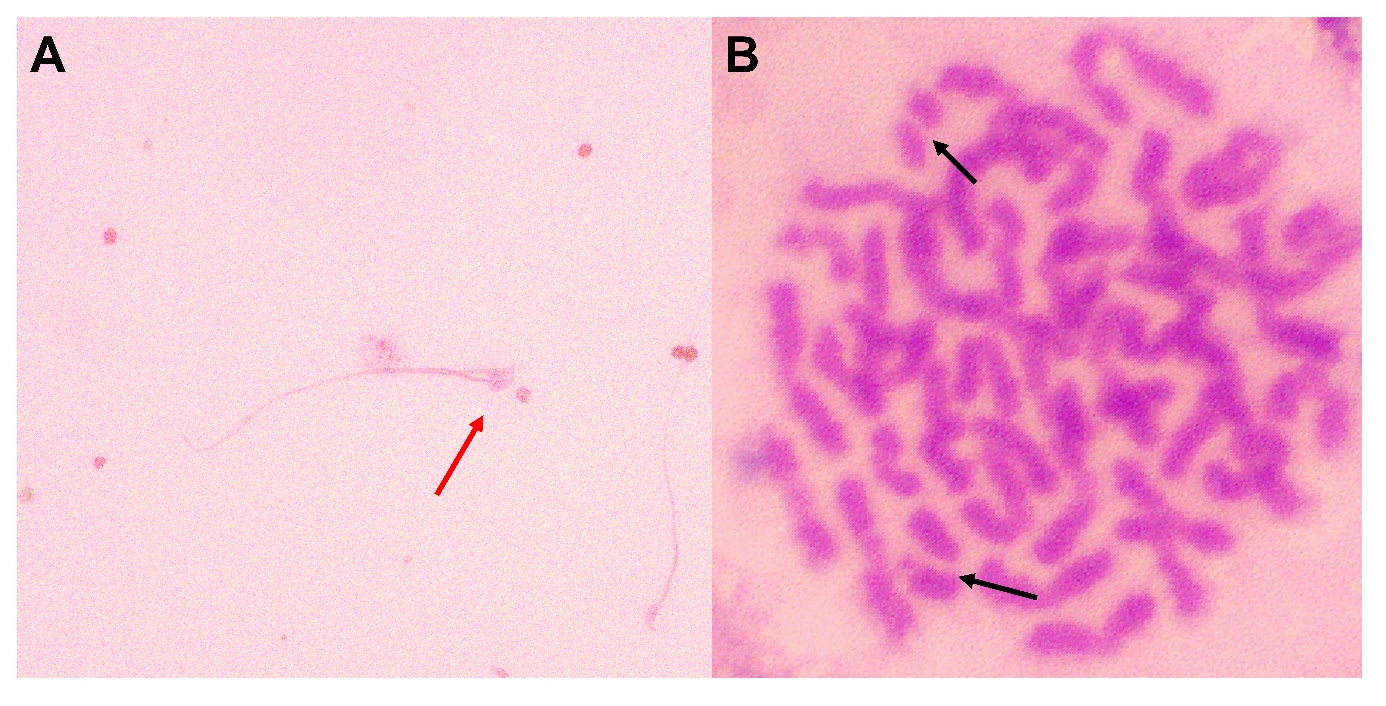


**Supplementary Figure 1.** Sperm and chromosomal teratogenic tissue sections. (A) Double-headed sperm found in mouse sperm malformation experiments (red arrow). (B) Chromosome breakage was found in the high dose group of chromosome aberrations (black arrow).


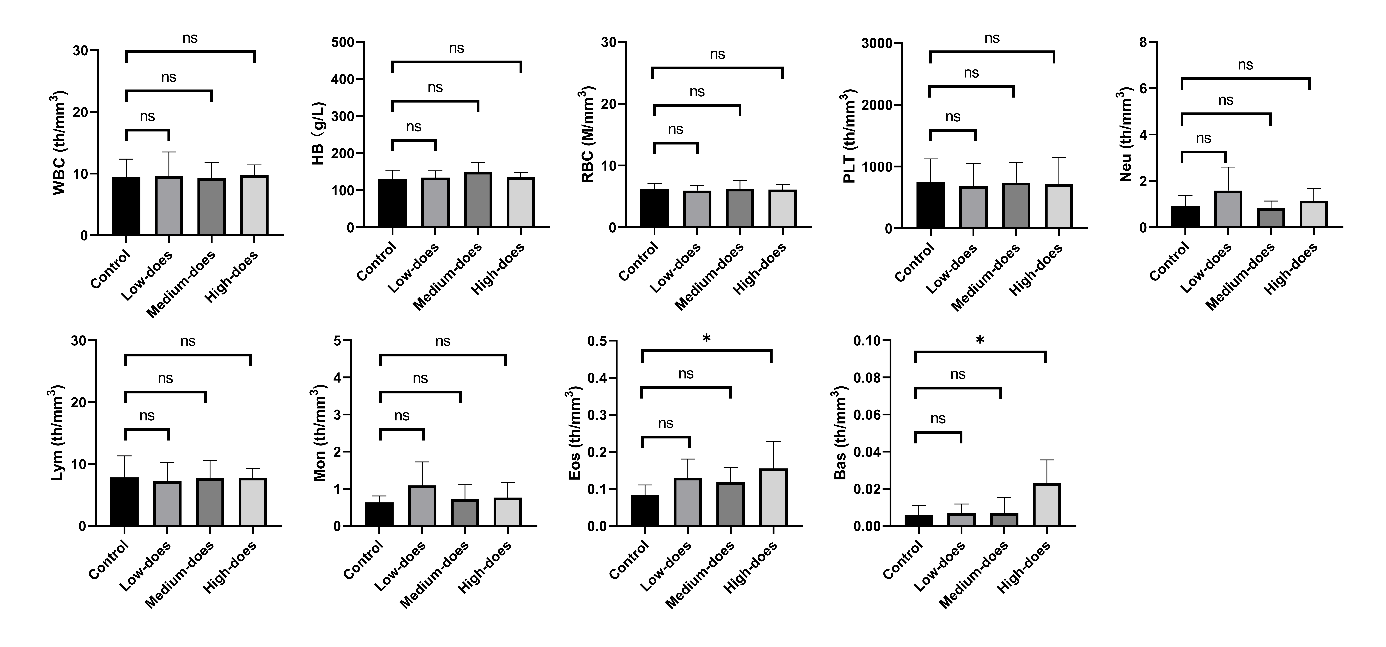


**Supplementary Figure 2.** Hematological parameters of 45-day subchronic toxicity test. WBC: White blood cell count; HB: Hemoglobin; RBC: Red blood cell count; PLT: Platelet count; Neu: Neutrophils; Lym: Lymphocytes; Mon: Monocytes; Eos: Eosinophils; Bas: Basophils.


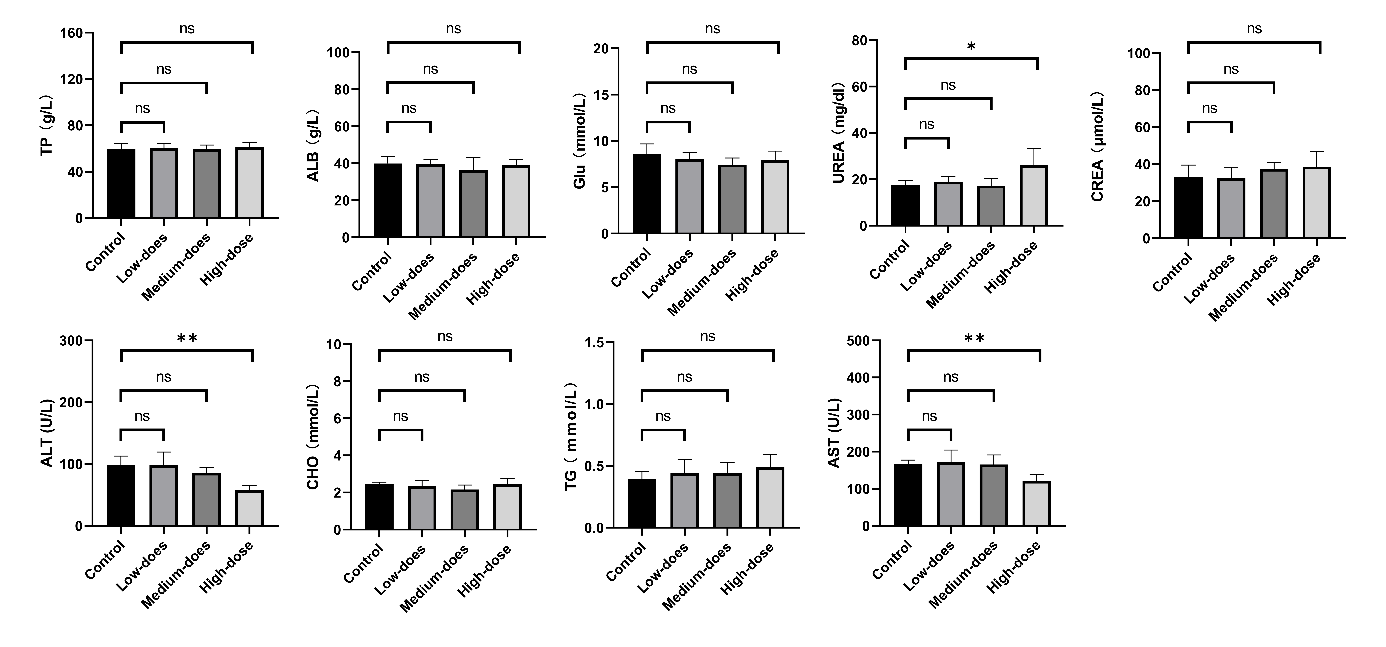


**Supplementary Figure 3.** Serum biochemical indicators of 45-day subchronic toxicity test. TP: Total protein; ALB: Albumin; Glu: Glucose; UREA: Urea; CREA: Creatinine; ALT: Alanine aminotransferase; CHO: Cholesterol; TG: Triglycerides; AST: Aspartate aminotransferase.


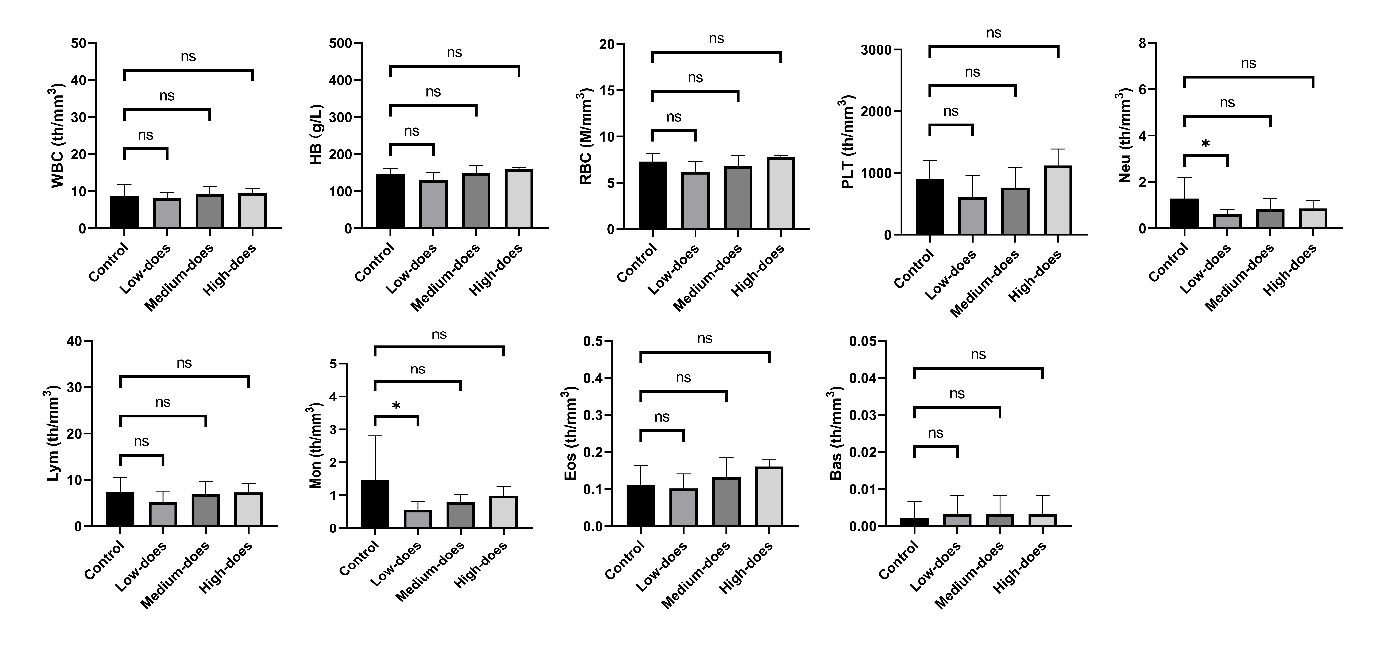


**Supplementary Figure 4.** Hematological parameters of 90-day subchronic toxicity test. WBC: White blood cell count; HB: Hemoglobin; RBC: Red blood cell count; PLT: Platelet count; Neu: Neutrophils; Lym: Lymphocytes; Mon: Monocytes; Eos: Eosinophils; Bas: Basophils.


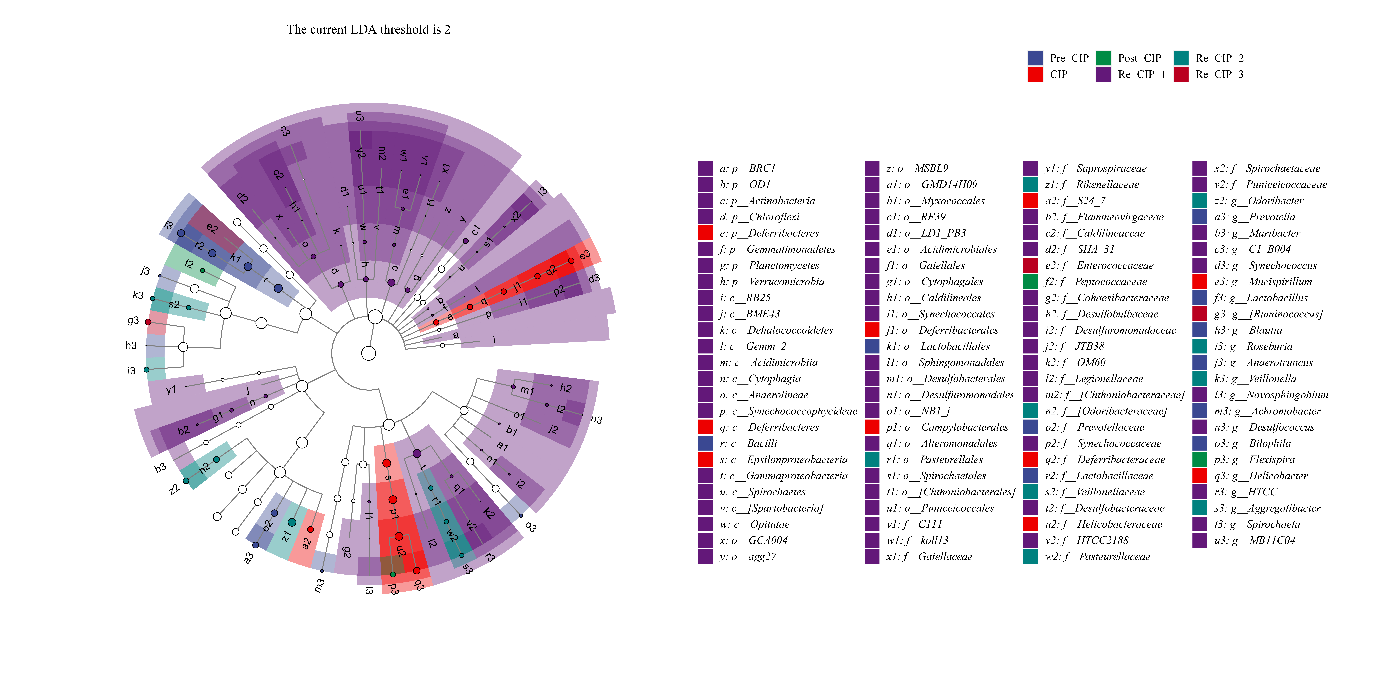


**Supplementary Figure 5.** Lefse analysis identified the microbes that showed significant differences in gut microbiota abundance at different time points of CIP treatment.
